# FOSTERING MULTIPARTNERSHIP IN DEVELOPING A DEMENTIA-FRIENDLY COMMUNITY WITH AN OPEN DEMENTIA DAY CENTER IN TAIWAN

**DOI:** 10.1093/geroni/igac059.1363

**Published:** 2022-12-20

**Authors:** Ling-Hui Chang, Men-An Pan, Li-Xue Wu, Chi-pang Lu, Liwen Sung, Pai--Chuan Huang

**Affiliations:** National Cheng Kung University, Tainan City, Tainan, Taiwan (Republic of China); Pingtung County Government, Pingtung City, Pingtung, Taiwan (Republic of China); Pingtung County Government, Pingtung City, Pingtung, Taiwan (Republic of China); Research Center For Humanities And Social Sciences, Tainan City, Tainan, Taiwan (Republic of China); Department of Architecture, Tainan City, Tainan, Taiwan (Republic of China); Occupational Therapy, College of Medicine, Tainan City, Tainan, Taiwan (Republic of China)

## Abstract

Introduction. Supporting persons with dementia without increasing the demands of formal care is an issue that many countries face today. Pingtung County proposed an innovative practice of an open dementia day center situated in a dementia-friendly community (DFC), where people with dementia in the center can freely go outside and enjoy community living. This presentation examines the experience of an university academic team in fostering a municipality-academic-community partnership as we tried to “flip” the care of a traditional dementia day center to adopt an “open” care model and concurrently to make the nearby community dementia-friendly. Method. A pragmatic research paradigm was adopted to address the socially situated situation in which we encountered when building a dementia-friendly community and the strategies we adopted to respond to the problem. Results/Discussion. Three themes: (1) Partnering with local government, e.g. to reduce the cognitive and physical barriers for outdoors activities and to increase the competence of local officials in DFC. (2) Partnering with local community and business: e.g. to increase the DFC awareness of community volunteers and to initiate a DFC business project, (3) building an academic team: e.g. to engage faculties who were motivated to pioneer an innovative practice, to fulfill their social responsibility, bridge the gap between theory, research, and practice. Conclusion. Significant changes were made in the community and the day center towards DFC. Community stigma towards dementia and community inertia was major obstacles. The allocation of city resources, personal drive towards DFC, and regular communication were key, despite the process was fraught with compromise, power dynamics, and conflicts of interests.

